# Combined Laparoscopic and Thoracoscopic Management of Gastrobronchial Fistula Developed Early After Laparoscopic Sleeve Gastrectomy: A Video Presentation

**DOI:** 10.1007/s11695-025-08051-9

**Published:** 2025-07-15

**Authors:** Mohamed Hany, Ahmed Mohamed Elazazy Mohamed Megahed, Ahmed Ibrahim Elraey, Mona S. Youssef, Mohamed Samir, Bart Torensma

**Affiliations:** 1 Department of Surgery, Medical Research Institute, Alexandria University, Alexandria, Egypt; 2 Ameryea General Hospital Egyptian Ministry of Health, Alexandria, Egypt; 3 Department of Clinical Epidemiology, Erasmus MC, Rotterdam, Netherlands; 4 Department of Surgery, Weight Works Clinics, Amersfoort, Netherlands

**Keywords:** Gastro bronchial fistula, Sleeve gastrectomy, Metabolic bariatric surgeries, Preoperative optimization

## Introduction

Metabolic bariatric surgeries (MBS) have gained widespread popularity as effective treatments for obesity and its related comorbidities. According to the 8th International Federation for the Surgery of Obesity and Metabolic Disorders (IFSO) global registry report, laparoscopic sleeve gastrectomy (LSG) is the most commonly performed procedure, accounting for 60.4% of cases, followed by Roux-en-Y gastric bypass (RYGB) at 29.5%, and one-anastomosis gastric bypass (OAGB) at 4.3% [1].

One of the most serious postoperative complications is the occurrence of leaks or fistulas. Gastric leaks have been reported in 2.4% of cases following LSG. Such leaks can result in subphrenic abscesses, which may progress to more severe complications, including pulmonary abscesses or gastrobronchial fistulas (GBFs) [2, 3].

GBF, a rare but severe complication, arises due to the irritant nature of gastric secretions, elevated gastric pressure, and the anatomical proximity of the stomach to the lungs. This condition presents significant management challenges and can seriously affect the patient. The global incidence of GBF remains unknown, but studies range from 0.2 to 0.4%, highlighting the need to identify its predisposing factors and to develop preventive, diagnostic, and therapeutic strategies [4–6].

Sakran et al.’s systematic review investigated gastric fistulas extending into the thoracic cavity following LSG. The review included 55 patients with a wide range of initial presentations, including vomiting, empyema, abdominal pain, lung abscess, and nonspecific symptoms. The most commonly reported manifestations were leaks, subphrenic collections, pneumonia, cough, and fever [7, 8].

Potential predisposing factors for GBF include recurrent undrained abscesses in the upper abdomen or early removal of surgical drains, which can lead to chronic subphrenic inflammation. However, due to the limited data on gastrobronchial fistulas, there are currently no established guidelines, standardized algorithms, or consensus on the clinical progression, timing, multidisciplinary management, or surgical treatment of this rare complication [9].

Several factors must be considered when managing GBF, including the patient’s nutritional and general health status, the adequacy of previous conservative treatments, local inflammatory conditions that may complicate surgical dissection, and any gastrobronchial communication [7, 8].

This video presentation aims to provide an in-depth understanding of GBF, a rare but potentially life-threatening complication following MBS. This includes exploring its clinical presentation, diagnostic challenges, and management strategies. By highlighting a specific case and reviewing existing literature, we emphasize the importance of a multidisciplinary approach, tailored treatment plans, and preoperative optimization in improving patient outcomes. Additionally, we seek to contribute to the discussion on standardized algorithms for managing this complex condition.

## Case Presentation

A 40-year-old male patient with a BMI of 55.3 kg/m^2^ (weight 160 kg, height 170 cm) was referred to our hospital in July 2024, 21 days after undergoing LSG. Symptoms began 1 week postoperatively, including vomiting, fever, left chest pain, and a persistent cough. Initial treatment at the referring facility included IV antibiotics, proton pump inhibitors, IV fluids, and antipyretics, but the patient showed no improvement. A computer tomography (CT) scan and upper endoscopy on postoperative day 14 confirmed a GBF, and the patient was transferred to our centre with more facilities.

Upon arrival, the patient presented with hypoxia (oxygen saturation 82% on room air), fever, tachypnea, tachycardia (heart rate 115 bpm), hypotension (blood pressure 100/50 mmHg), hemoptysis, and a wheezy left chest. Laboratory findings showed anemia (hemoglobin 8.4 g/dL), leukocytosis (WBC 16,000 cells/μL), elevated CRP (440 mg/dL), and renal impairment (urea 111 mg/dL; creatinine 2 mg/dL). The patient was admitted to the intensive care unit (ICU) for multidisciplinary management, including infection control, nutritional optimization, and respiratory support (full nutritional management phases on the ICU overview and laboratory evidence of nutritional improvement, Appendix 1).

The endoscopic evaluation revealed a gastric fistula at the upper part of the gastric sleeve with a fistulous tract extending through the diaphragm and a larger defect distal to the initial site. CT imaging confirmed the passage of contrast into the left lower lobe bronchi, resulting in hydropneumothorax and basal segment atelectasis. Treatment included the placement of a 23 cm covered metallic mega stent, a pigtail catheter inserted in the left chest collection, and the gradual reintroduction of oral fluids and enteral feeding.

After 2 weeks in the ICU and 2 weeks on the ward, the patient stabilized, and the pigtail catheter was removed. However, fever, cough, sputum discharge, and vomiting persisted despite targeted antibiotic therapy for Klebsiella identified in the drainage cultures. Upper endoscopic gastro-duodenoscopy (EGD) showed stent migration and pus around the stent, suggesting poor fistula healing. One month after stenting, and improving laboratory results (Appendix 1), a laparoscopic exploration was planned due to ongoing symptoms.

Intraoperative findings included disruption of the upper part of the gastric sleeve, an abscess cavity with diaphragmatic communication to the lung, and a second fistulous tract near the antrum.

A total gastrectomy and Roux-en-Y esophagojejunostomy were performed using a 25 mm circular stapler for the alimentary limb (100 cm) and biliary limb (60 cm), with the closure of the diaphragmatic defect. Thoracoscopic decortication addressed lung adhesions, drained a parenchymal abscess, and resected damaged lung tissue. Postoperative EGD confirmed no leaks, and a nasojejunal feeding tube and drain were placed.

The patient was extubated the day after surgery. Over the following weeks, he progressed from nasojejunal feeding to oral intake, with gradual recovery. CT scans showed no leaks, and the chest and abdominal drains were removed. The patient was discharged 1 month post-surgery, with a total hospital stay of approximately 8 weeks. Satisfactory progress was noted at follow-up, including a weight reduction to 144 kg. Now, ± 8 months post-discharge, the follow-up shows that no late complications have occurred. The patient’s weight was 119 kg at 6 months and 110 kg at 8 months; he is taking multivitamins.

Our key contributions include the following: (1) *timely management*—early recognition and prompt intervention; (2) *multidisciplinary team approach*—coordinated care between gastroenterology, thoracic surgery, interventional radiology, and nutrition; (3) *individualized treatment*—tailored approach based on patient’s condition and response; (4) *combined approaches*—integration of endoscopic, radiological, and surgical interventions in a staged manner.

## Conclusion

Gastrobronchial fistulas (GBF) treatment management strategies must be individualized through a collaborative, multidisciplinary approach for each case. Early efforts should prioritize controlling local sepsis using non-surgical and endoscopic methods. The appropriate timing for surgical intervention remains uncertain, relying on the patient’s stability and the duration between the primary surgery and the onset of the leak.

## Supplementary Information

Below is the link to the electronic supplementary material.

Supplementary file1 (DOCX 16 KB)

Supplementary video (MP4 223 MB)

## Data Availability

No datasets were generated or analysed during the current study.
