# Correction to: Combined Laparoscopic and Thoracoscopic Management of Gastrobronchial Fistula Developed Early After Laparoscopic Sleeve Gastrectomy: A Video Presentation

**DOI:** 10.1007/s11695-025-08112-z

**Published:** 2025-08-16

**Authors:** Mohamed Hany, Ahmed Mohamed Elazazy Mohamed Megahed, Ahmed Ibrahim Elraey, Mona S. Youssef, Mohamed Samir, Bart Torensma

**Affiliations:** 1 Department of Surgery, Medical Research Institute, Alexandria University, Alexandria, Egypt; 2 Ameryea General Hospital Egyptian Ministry of Health, Alexandria, Egypt; 3 Department of Clinical Epidemiology, Erasmus MC, Rotterdam, Netherlands; 4 Department of Surgery, Weight Works Clinics, Amersfoort, Netherlands

**Correction to: Obesity Surgery**

10.1007/s11695-025-08051-9

The original article was revised to add the missing video file.

## Supplementary Information

Below is the link to the electronic supplementary material.

Supplementary file1 (MP4 228635 KB)

