# Supplementary material for: Combined Laparoscopic and Thoracoscopic Management of Gastrobronchial Fistula Developed Early After Laparoscopic Sleeve Gastrectomy: A Video Presentation
Source: Obes Surg. 2025 Jul 15;35(9):3949–51. doi: 10.1007/s11695-025-08051-9 (PMC12457462; doi:10.1007/s11695-025-08051-9)
Supplement: Supplementary file 1 — (DOCX 16 KB) [file 11695_2025_8051_MOESM1_ESM.docx]

**Appendix 1: Nutritional Management Phases Overview and Laboratory Evidence of Nutritional Improvement.**

**Nutritional Management Phases Overview:**

**Phase 1 (Week 1): Stabilization & Clear Liquids** - IV fluid support with thiamine supplementation to prevent Wernicke's encephalopathy. Gradual introduction of clear liquids (sugar-free broths, electrolyte solutions) progressing to full liquids with protein supplements. Target: 60-80g protein/day from liquid sources.

**Phase 2 (Week 2): Pureed Diet Advancement** - Introduction of pureed foods (pureed lean meats, eggs, soft fish, cottage cheese) while maintaining liquid protein supplements. Micronutrient repletion initiated with oral iron, B12 injections, and bariatric multivitamins. Target: 100g protein/day, ~1200-1500 kcal/day.

**Phase 3 (Week 3): Soft Solid Foods** - Progression to soft, minced foods requiring gentle chewing. Continued emphasis on protein-first approach with tender meats, soft vegetables, and appropriate textures. Target: 120g protein/day, ~1500-1800 kcal/day.

**Phase 4 (Week 4): Pre-Surgery Optimization** - Maintained soft solid diet with possible texture liberalization. Focus on achieving optimal nutritional parameters before surgery. Final supplementation and immunonutrition support.

Key improvements were achieved through: (1) **Inflammation control** - stent placement and antibiotics reduced sepsis, allowing the shift from catabolism to anabolism as evidenced by decreasing CRP levels; (2) **Protein repletion** - adequate protein intake (140-175g/day) met stress-induced requirements, supporting tissue repair and immune function; (3) **Micronutrient correction** - iron, B12, folate, and zinc supplementation addressed deficiencies and supported erythropoiesis and wound healing; (4) **Metabolic optimization** - controlled glucose levels and correction of electrolyte imbalances facilitated anabolic processes.

Within one month, we observed measurable improvements: hemoglobin increased from 8.4 to >12 g/dL, albumin trended upward as CRP decreased (reflecting reduced inflammation and improved protein synthesis), and the patient demonstrated better functional status. This comprehensive approach, supported by evidence-based critical care nutrition guidelines for obese patients, successfully prepared the patient for definitive surgical intervention.

**Laboratory Evidence of Nutritional Improvement:**

| **Parameter** | **Baseline (Post-Sepsis)** | **Week 1** | **Week 2** | **Week 4** |
| --- | --- | --- | --- | --- |
| **Weight (kg)** | 160 kg | 158-160 kg (slight loss of fluid weight) | 157 kg | 156 kg |
| **Hemoglobin (g/dL)** | 8.4 (pre-transfusion); 11.0 after transfusion | 11.0 | 11.5 | 12.0 |
| **Albumin (g/dL)** | 2.2 g/dL (low) | 2.3 | 2.5 | 2.8-3.0 |
| **CRP (mg/L)** | 100 mg/L (peak) | 80 | 50 | 20 |

These serial measurements demonstrate: (1) **Hemoglobin improvement** from 8.4 to 12.0 g/dL through iron and B12 supplementation and reduced inflammation; (2) **Albumin recovery** from 2.2 to 2.8-3.0 g/dL, reflecting improved protein synthesis as inflammation subsided; (3) **CRP normalization** from 100 to 20 mg/L, indicating resolution of sepsis and inflammatory state; (4) **Controlled weight loss** primarily from fluid loss and fat reduction while preserving lean mass through high-protein nutrition.
